# Non‐tumour bone marrow lymphocytes correlate with improved overall survival in childhood acute lymphoblastic leukaemia

**DOI:** 10.1002/pbc.26093

**Published:** 2016-06-27

**Authors:** Claire Edwin, Joanne Dean, Laura Bonnett, Kate Phillips, Russell Keenan

**Affiliations:** ^1^ Department of Women's and Children's HealthInstitute of Translational MedicineUniversity of LiverpoolLiverpoolUK; ^2^ Department of HaematologyAlder Hey Children's NHS Foundation TrustEaton RoadLiverpoolUK; ^3^ Department of BiostatisticsInstitute of Translational MedicineUniversity of LiverpoolUK; ^4^ Department of Haematology and OncologyAlder Hey Children's NHS Foundation TrustEaton RoadLiverpoolUK

**Keywords:** acute lymphoblastic leukaemia, immunophenotype, lymphocyte, overall survival, paediatric cancer

## Abstract

Composition of tumour immune cell infiltrates correlates with response to treatment and overall survival (OS) in several cancer settings. We retrospectively examined immune cells present in diagnostic bone marrow aspirates from paediatric patients with B‐cell acute lymphoblastic leukaemia. Our analysis identified a sub‐group (∼30% of patients) with >2.37% CD20 and >6.05% CD7 expression, which had 100% OS, and a sub‐group (∼30% of patients) with ≤2.37% CD20 and ≤6.05% CD7 expression at increased risk of treatment failure (66.7% OS, *P* < 0.05). Immune cell infiltrate at diagnosis may predict treatment response and could provide a means to enhance immediate treatment risk stratification.

**ABBREVIATIONS**
ALL: acute lymphoblastic leukaemia
BM: bone marrow
CD: cluster of differentiation
LFS: leukaemia‐free survival
NK: natural killer
OS: overall survival

## 1 INTRODUCTION

Conventional chemotherapeutic regimens cure a significant proportion of paediatric patients with B‐cell acute lymphoblastic leukaemia (B‐ALL). Improvements in survival rates observed over recent decades can be attributed to the introduction of efficient testing for minimal residual disease during treatment and new combinations of chemotherapeutics. However, considerable adverse effects are associated with regimens used to treat paediatric B‐ALL. Strategies to identify patients in whom lower dose treatment would be clinically effective or treatment intensification would enhance survival, represent attractive avenues to reduce long‐term cytotoxicity and/or enhance overall survival (OS).

Recent advances in our understanding of tumour immunology suggest that an individual's immune response, pre‐therapy against their own tumour, significantly influences disease progression.^1^ Several parameters associated with the composition of immune infiltrate in solid tumours have been shown to correlate with prognosis, and in some instances, to predict patient survival more accurately than any other parameter.^2^, ^3^ In B‐ALL, two recent reports examining the immunological composition of bone marrow (BM) at diagnosis demonstrate correlation between CD4^+^ (where CD is cluster of differentiation) T lymphocytes and favourable early response in paediatric patients 4 and CD8^+^ T lymphocytes and improved OS in adult patients.^5^ In this study, we examined the composition at diagnosis of non‐malignant lymphocytes in the BM of paediatric patients with B‐ALL. We aimed to identify whether non‐malignant lymphocytes routinely measured in BM aspirate by flow cytometry are associated with OS.

## 2 METHODS AND RESULTS

We reviewed the medical records of 153 children diagnosed with ALL at Alder Hey Children's Hospital, Liverpool, between 2002 and 2009 in accordance with NHS Health Research Authority and Royal College of Pathologists’ guidelines. All patients had been treated in accordance with UKALL 97/99 or UKALL 2003 trial protocol. In 55 cases, flow cytometry data from clinical diagnostic BM aspirate could be recovered in List Mode data format and re‐analysed to enumerate data related to the non‐tumour cells present. All 55 patients analysed were diagnosed with common ALL (*de novo* precursor B‐ALL), patient characteristics are shown in Table 1. Patients with Philadelphia chromosome positive B‐ALL, common ALL with aberrant CD20 tumour expression or Down syndrome were excluded from this study. There were eight deaths and the remaining 47 patients were alive and well at the time of last follow‐up, although four had experienced relapse, but were treated successfully with salvage chemotherapy.

**Table 1:** Patient characteristics

	Study cohort, n = 55	Total patients, n = 153
Median age in years, n (IQR)	5.0 (2.5–7.5)	4.0 (1.5–6.5)
Male gender, n (%)	30 (54.5)	83 (54.2)
Age groups		
<10 years, n (%)	44 (80.0)	125 (81.7)
≥10 years, n (%)	11 (20.0)	28 (18.3)
WCC at diagnosis		
<50 × 10^9^ l^−1^, n (%)	46 (83.6)	129 (84.3)
≥50 × 10^9^ l^−1^, n (%)	9 (16.4)	24 (15.7)
Initial treatment protocol		
Regimen A (low risk), n (%)	36 (65.5)	103 (67.3)
Regimen B (high risk), n (%)	19 (34.5)	50 (32.7)

IQR, inter‐quartile range; WCC, white cell count.

**Figure 1:**
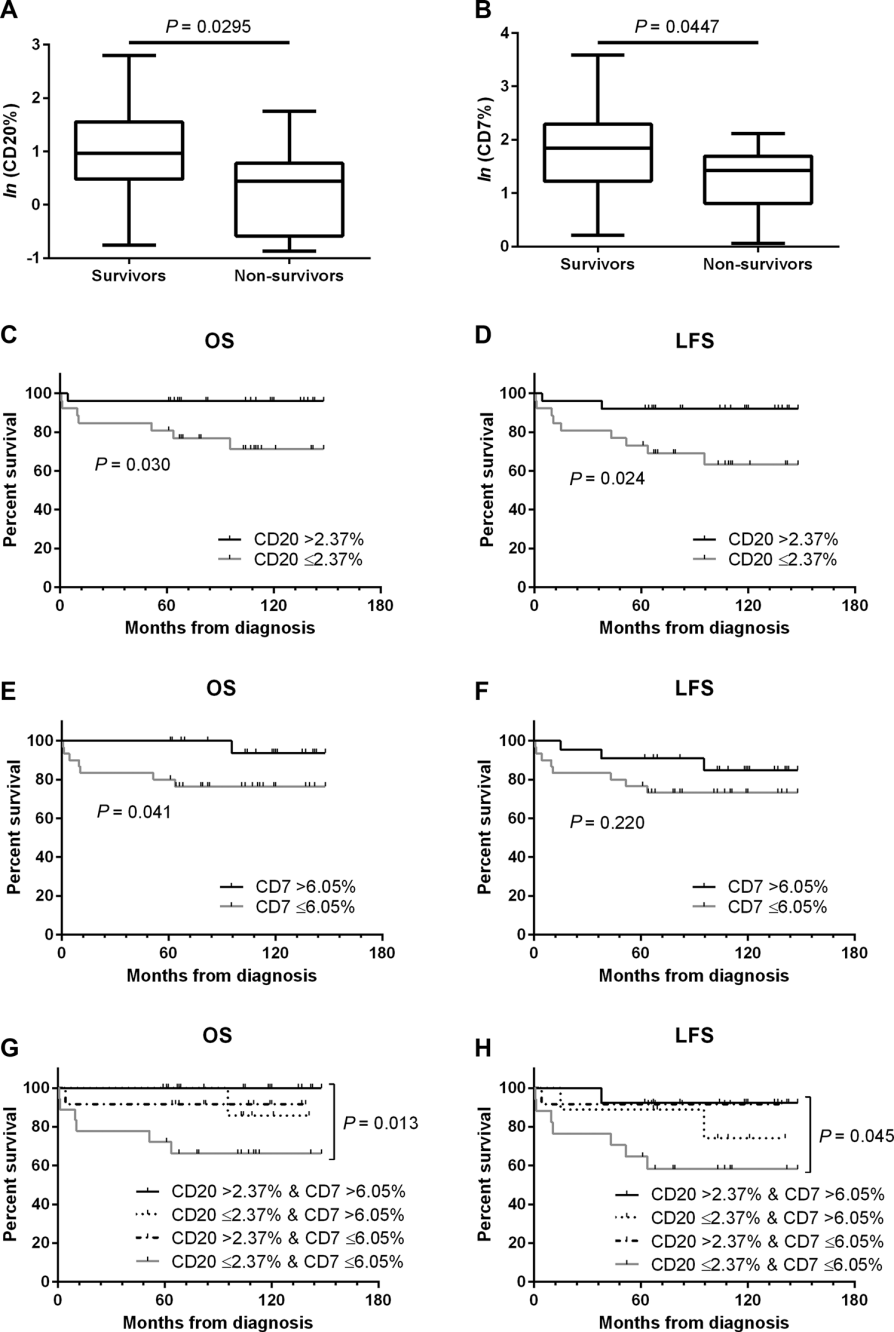
CD20^+^ lymphocytes were increased in survivors compared to non‐survivors (A), CD7^+^ lymphocytes were increased in survivors compared to non‐survivors (B). Enhanced OS (C) and LFS (D) was observed in paediatric patients with B‐ALL having >2.37% CD20^+^ lymphocytes. Enhanced OS (E) but not LFS (F) was observed in paediatric patients with B‐ALL having >6.05% CD7^+^ lymphocytes. 100% OS was observed in patients having >2.37% CD20^+^ and >6.05% CD7^+^ lymphocytes (G). 87.5% LFS was observed in patients having >2.37% CD20^+^ and >6.05% CD7^+^ lymphocytes compared to 61.1% LFS in patients having ≤2.37% CD20 and ≤6.05% CD7 expression (H).

We assessed the measured relative frequency values of CD markers in diagnostic BM aspirate between survivors and non‐survivors. Tumour lymphocytes (CD19^+^, CD10^+^) and non‐malignant B‐lymphocytes (CD19^+^, CD10^−^) were equivalent between study groups; however, an increased relative frequency of the mature B‐lymphocyte marker, CD20, was observed in survivors (*P* = 0.0295; Fig. 1A). T lineage cells (CD2^+^) were equivalent between study groups; however, an increased relative frequency of the mature T‐lymphocyte and natural killer (NK) cell marker, CD7, was observed in survivors (*P* = 0.0447; Fig. 1B). Following associated receiver operating characteristic analysis, cut‐offs of 2.37% CD20 expression (sensitivity 88%, specificity 56%) and 6.05% CD7 expression (sensitivity 88%, specificity 53%) were selected to differentiate between patients with high and low expression.

Comparing patients with high CD20 expression (>2.37%; n = 28) to the patients with lower expression (≤2.37%; n = 27), we observed significantly increased OS of 96.4% compared to 74.1% (*P* = 0.030; Fig. 1C), and improved leukaemia‐free survival (LFS) of 92.9% compared to 66.7% (*P* = 0.024; Fig. 1D). In a similar analysis of CD7, we observed increased OS of 96.0% in patients with high CD7 expression (>6.05%; n = 25) compared to 76.7% in the patients with low expression (≤6.05%; n = 30; *P* = 0.041; Fig. 1E), although no improvement in LFS was noted (Fig. 1F). Combining these factors, we observed that patients with high CD20 expression and high CD7 expression (n = 16) had 100% OS compared to 66.7% in patients with low expression of both markers (n = 18; Fig. 1G; *P* = 0.013), and improved LFS of 87.5% compared to 61.1% (*P* = 0.045; Fig. 1H).

## 3 DISCUSSION

Our retrospective analysis of CD marker expression in diagnostic BM aspirates identified a group of patients (16 out of 55) who could be characterised at diagnosis according to high CD20 and high CD7 expression, who experienced 100% OS. Conversely, patients who could be characterised according to low CD20 and low CD7 expression (18 out of 55) experienced significantly reduced OS, 66.7%, suggesting that the BM immune infiltrate at diagnosis is indicative of, or can be correlated with, their response to treatment.

An increasing body of literature exists linking immune system parameters at diagnosis with prognosis in cancer patients.^1^ Immune cell infiltrate may represent an ongoing but ineffective antitumour immune response, or a collection of tumour‐promoting cells recruited into the tumour micro‐environment. This so‐called ‘tumour immune contexture’ may be relevant to understanding a patients’ response to treatment,^6^ and the induction of an antitumour immune response has the potential to enhance survival prospects. Lymphocytes perform immune surveillance and may recognise malignant cells as immunogenic.^7^ CD20 is expressed on mature B cells with the exception of terminally differentiated plasmablasts or plasma cells. Resting B cells express surface immunoglobulin (Ig) as a receptor;^8^ antigen engagement of surface Ig can activate B cells and leads to internalisation of antigen and presentation of peptides complexed with major histocompatibility complex class II at the cell surface for interaction with CD4^+^ T cells.^8^, ^9^ Lymphocytes are also immune effector cells, and may mediate tumour cell death through granule exocytosis or death receptor signalling, cytotoxic T cells and NKs are key effectors in these mechanisms. Interaction between B and T cells serves to augment adaptive immune responses through cross‐priming of T cells, reciprocal enhancement of activation signalling in each cell type and the production of immunostimulatory cytokines.^9^, ^10^ Thus, the collaboration of different types of lymphocyte may confer the capacity to develop a robust adaptive immune response against autologous tumour cells.

Our findings, combined with the findings reported by Lustfeld et al.,^4^ provide the first information related to the relevance of the tumour immune contexture in paediatric ALL. An increased frequency of mature immune cell components within the BM tumour micro‐environment is strongly associated with both early treatment response^4^ and successful chemotherapeutic treatment. Further, elucidating the relationships between immune infiltrate components at diagnosis and treatment response may provide a means to enhance immediate treatment risk stratification. Of the eight children who died in this study, three had no clinical high risk features and were treated according to Regimen A. All three of these children had low CD20 levels at diagnosis, and two of these three also had low CD7 levels. Reliable identification of very good risk and very high risk patients at diagnosis potentially offers the opportunity to reduce or intensify therapy from induction onward. Our data clearly define two important patient sub‐groups; firstly, one for which the chemotherapeutic treatment regimen was 100% successful, and secondly, one in which patients were at significantly increased risk of treatment failure. Notwithstanding the limitations of this single centre retrospective study of small numbers, it seems relevant that the tumour immune contexture in paediatric ALL receives future attention, both to confirm our observations and to investigate mechanistically the benefit to patients of mature immune cell BM infiltrate at treatment commencement.
